# Treatment Outcomes in Global Systematic Review and Patient Meta-Analysis of Children with Extensively Drug-Resistant Tuberculosis

**DOI:** 10.3201/eid2503.180852

**Published:** 2019-03

**Authors:** Muhammad Osman, Elizabeth P. Harausz, Anthony J. Garcia-Prats, H. Simon Schaaf, Brittany K. Moore, Robert M. Hicks, Jay Achar, Farhana Amanullah, Pennan Barry, Mercedes Becerra, Domnica I. Chiotan, Peter C. Drobac, Jennifer Flood, Jennifer Furin, Medea Gegia, Petros Isaakidis, Andrei Mariandyshev, Iveta Ozere, N. Sarita Shah, Alena Skrahina, Elena Yablokova, James A. Seddon, Anneke C. Hesseling, for The Collaborative Group for Meta-Analysis of Paediatric Individual Patient Data in MDR TB

**Affiliations:** Stellenbosch University, Cape Town, South Africa (M. Osman, A.J. Garcia-Prats, H.S. Schaaf, J.A. Seddon, A.C. Hesseling);; State University of New York Upstate Medical University, Syracuse, New York, USA (E.P. Harausz);; Centers for Disease Control and Prevention, Atlanta, Georgia, USA (B.K. Moore);; University of California, San Francisco, California, USA (R.M. Hicks);; Médecins Sans Frontières, London, UK (J. Achar); Indus Hospital, Karachi, Pakistan (F. Amanullah);; California Department of Public Health, Richmond, California, USA (P. Barry, J. Flood);; Harvard Medical School, Boston, Massachusetts, USA (M. Becerra, P.C. Drobac, J. Furin);; Romanian National TB Program, Bucharest, Romania (D.I. Chiotan);; World Health Organization, Geneva, Switzerland (M. Gegia); Médecins Sans Frontières, Mumbai, India (P. Isaakidis);; Northern State Medical University, Arkhangelsk, Russia (A. Mariandyshev, E. Yablokova); Riga Eastern Clinical University Hospital, Riga, Latvia (I. Ozere);; Albert Einstein College of Medicine, Bronx, New York, USA (N.S. Shah);; Republican Research and Practical Centre for Pulmonology and TB, Minsk, Belarus (A. Skrahina); Imperial College London, London (J.A. Seddon)

**Keywords:** tuberculosis and other mycobacteria, tuberculosis, TB, extensively drug-resistant tuberculosis, XDR TB, multidrug-resistant TB, MDR TB, bacteria, antimicrobial resistance, outcomes, respiratory infections, mortality, meta-analysis, treatment outcomes, global systematic review, children

## Abstract

Extensively drug-resistant tuberculosis (XDR TB) has extremely poor treatment outcomes in adults. Limited data are available for children. We report on clinical manifestations, treatment, and outcomes for 37 children (<15 years of age) with bacteriologically confirmed XDR TB in 11 countries. These patients were managed during 1999–2013. For the 37 children, median age was 11 years, 32 (87%) had pulmonary TB, and 29 had a recorded HIV status; 7 (24%) were infected with HIV. Median treatment duration was 7.0 months for the intensive phase and 12.2 months for the continuation phase. Thirty (81%) children had favorable treatment outcomes. Four (11%) died, 1 (3%) failed treatment, and 2 (5%) did not complete treatment. We found a high proportion of favorable treatment outcomes among children, with mortality rates markedly lower than for adults. Regimens and duration of treatment varied considerably. Evaluation of new regimens in children is required.

## Abstract 2

Children had better treatment outcomes and lower mortality rates than adults.

Extensively drug-resistant tuberculosis (XDR TB) is a public health emergency that threatens global TB control. Multidrug-resistant TB (MDR TB) is caused by *Mycobacterium tuberculosis* that shows resistance to isoniazid and rifampin, and XDR TB includes additional resistance to any fluoroquinolone and a second-line injectable drug (*1*). In 2016, a total of 8,014 cases of XDR TB were reported to the World Health Organization (WHO) by 72 countries (*2*). Treatment success rates for XDR TB remain poor; only 30% of patients show cure or treatment completion, and costs for care far exceed those for drug-susceptible TB (*2*). There is an increasing awareness that children are also affected by MDR TB and XDR TB. Modeling studies estimated that as many as 2 million children currently have MDR TB globally, and MDR TB develops in an estimated 30,000 children <15 years of age each year (*3*,*4*). Estimates suggest that of children with MDR TB, 4.7% have XDR TB (*4*).

We recently completed a systematic review and individual patient data (IPD) meta-analysis commissioned by WHO that described clinical presentation, treatment, and outcomes for children treated for MDR TB (*5*). We reported data for 975 children with MDR TB from 18 countries; 731 (75%) had bacteriologically confirmed MDR TB, and 244 (25%) had clinically diagnosed MDR TB (*6*). Overall, 764 (78%) of 975 children had a successful treatment outcome (*6*), as defined by WHO guidelines (*7*). This meta-analysis provided information on pediatric aspects for the revised 2016 WHO drug-resistant TB treatment guidelines, and specific recommendations were subsequently made for treatment of MDR TB in children (*5*). Children with confirmed XDR TB were excluded because they were a distinct subgroup and insufficient evidence was available to make treatment recommendations for children with XDR TB at that time (*5*).

The management of XDR TB in children is challenging because of the limited availability of new drugs and appropriate treatment regimens. XDR TB treatment regimens for children have historically been individualized on the basis of mycobacterial drug-susceptibility testing (DST) of the organism of the child or the putative source case (*5*). There are limited data on the optimal combination of medications and the duration of treatment for XDR TB and major research gaps remain (*5*). Therefore, we aimed to describe clinical manifestations, routine treatment, and outcomes for children with confirmed XDR TB in the era preceding access to novel anti-TB drugs for children.

## Methods

### Data Collection

As part of a systematic review and IPD meta-analysis, we collected data from global collaborators on children <15 years of age treated for MDR TB as part of a defined treatment cohort (*6*). We identified published and unpublished data from retrospective and prospective studies by using a broad search strategy. Eligible studies were identified, and individual level patient data were requested from each author by using a standardized data collection tool. We requested demographics, clinical details, and outcomes on the basis of specified definitions. Additional interpretation of data was conducted by the study team, and the primary authors were contacted to resolve queries. Data were obtained for 1,012 children treated during 1999–2013 (*6*). Detailed methods and outcomes of IPD meta-analysis for 975 children with MDR TB and pre-XDR TB (MDR TB with additional resistance to either a fluoroquinolone or an injectable drug, but not both) was reported (*6*). We report on children identified through systematic review of drug-resistant TB in children who had bacteriologically confirmed XDR TB. Therefore, this analysis only included children who were investigated for TB, had presence of *M. tuberculosis* confirmed bacteriologically, had isolates tested for resistance to second-line anti-TB drugs, and received a treatment outcome during the episode of TB.

### Definitions

We defined TB as pulmonary TB when disease was localized to the lungs or intrathoracic lymph nodes and as extrapulmonary TB when disease was found at site distant from the lungs, including pleural effusions and miliary TB. We classified pulmonary TB as severe or nonsevere by using adapted criteria of Wiseman et al. (*8*) on the basis of a review of reported chest radiographs by 2 independent reviewers; a third reviewer arbitrated discordance. Previous TB treatment history and type of treatment previously received (for drug susceptible TB or drug-resistant TB), was documented when known. We defined malnutrition as being underweight for age (weight-for-age z-score <–2) or per the report of the treating clinician.

Because the review spanned many years and sites, DST methods varied by region and period. Data collected for treatment of XDR TB varied, and sites inconsistently submitted data on individual drugs used and drug dose or duration of treatment. We report intensive phase and continuation phase as submitted, for which investigators defined these 2 stages according to their clinical practice. The intensive phase typically refers to initial months of treatment, which include more drugs and the use of an injectable drug. The continuation phase refers to a second phase of treatment generally with a reduction in the number of drugs.

We defined TB treatment outcomes by using standard 2014 WHO MDR TB outcome definitions as classified by treating clinicians: cure (treatment completed as recommended by the national policy without evidence of failure and >3 consecutive cultures taken at least 30 days apart were negative after the intensive phase of treatment); treatment complete (as for cure but without records of negative cultures); treatment failed (treatment stopped or requiring change of 2 drugs because of persistent positive cultures at end of the intensive phase or reversion to positive cultures in the continuation phase, or evidence of additional acquired resistance or adverse drug reactions); death (for any reason while receiving treatment); or loss to follow-up (treatment interruption for 2 consecutive months) (*9*). We defined favorable (cure and completed treatment) and unfavorable (treatment failure, death, or loss to follow-up) outcomes. Adverse events were inconsistently included in primary data and are not reported here.

### Statistical Analysis

We completed descriptive statistics for demographic and clinical variables. Missing data were noted, and each analysis reflects the sample size used. Categorical variables are presented as frequencies and percentages, and continuous variables (duration of intensive or continuation phase) are presented as median and interquartile ranges (IQRs). Age of children was categorized as <2 years, 2–4 years, 5–9 years, or 10–14 years. We used logistic regression with a preset 95% level of significance and calculated odds ratios (ORs) and 95% CIs to estimate and predict unfavorable outcomes. Children with unknown outcomes or loss to follow-up were excluded from regression analyses. We analyzed by using SAS software version 9.4 (https://www.sas.com).

### Ethical Approval

The study protocol was approved by the Health Research Ethics Committee of the Faculty of Medicine and Health Sciences, Stellenbosch University (reference no. X14/09/020). The oversight committee at the location institution of each contributor approved collection of data and submission to the collaborative systematic review.

## Results

Of 1,012 children included in the larger systematic review of children with MDR TB, 37 children from 11 countries had bacteriologically confirmed XDR TB (Table 1). We also compiled demographic and clinical characteristics at baseline (Table 2). Median age was 11 years (IQR 6.0–13.1 years). Thirty-two (87%) children had pulmonary TB only. Among children with pulmonary TB and chest radiographic findings, 20 (65%) of 31 had markers of severity, including disseminated/extensive disease, airway compression with lobar collapse, miliary opacification, or cavitation. HIV status was documented for 29 (78%) children; of these, 7 (24%) were infected with HIV.

**Table 1:** Overview of 14 studies and 37 children with confirmed cases of extensively drug-resistant tuberculosis*

Reference	Country	No. persons in study	No. children with XDR TB	Study design
Amanullah, unpub. data	Pakistan	29	1	Retrospective cohort
Banerjee et al. (*10*)	USA	18	1	Retrospective cohort
Chiotan, unpub. data	Romania	17	1	Retrospective cohort
Drobac et al. (*11*)	Peru	36	4	Retrospective cohort
Gegia et al. (*12*)	Georgia	55	3	Retrospective cohort
Hicks et al. (*13*)	South Africa	82	5	Retrospective cohort
Isaakidis et al. (*14*)	India	8	2	Retrospective cohort
Kuksa et al. (*15*)	Latvia	53	4	Retrospective cohort
Smirnova et al. (*16*)	Russia	38	1	Retrospective cohort
Moore et al. (*17*)	South Africa	339	5	Retrospective cohort
Seddon et al. (*18*)	South Africa	88	5	Retrospective cohort
Seddon et al. (*19*)	South Africa	131	2	Prospective cohort
Skrahina, unpub. data	Belarus	5	2	Retrospective cohort
Swaminathan et al. (*20*)	Tajikistan	27	1	Retrospective cohort

*Each study population involved all consecutively presenting bacteriologically confirmed and clinically diagnosed patients. XDR TB, extensively drug-resistant tuberculosis.

**Table 2:** Demographic and clinical characteristics of 37 children with bacteriologically confirmed extensively drug-resistant tuberculosis*

Characteristic	No. (%)
Age, y, median 11 y (IQR 6.0–13.1 y)	
<2	6 (16)
2–4	3 (8)
5–9 y	6 (16)
10–14 y	22 (60)
Sex	
F	23 (62)
M	14 (38)
Site of disease	
PTB	32 (86)
EPTB†	2 (5)
PTB and EPTB‡	3 (8)
Severe TB disease by chest radiography, n = 31§	
No	11 (35)
Yes	20 (65)
Documented adult TB source case, n = 28	
No	8 (29)
Yes	20 (71)
HIV status	
Uninfected	22 (59)
Infected	7 (19)
Unknown	8 (22)
Antiretroviral treatment	
Receiving treatment¶	7 (100)
Malnutrition, n = 30#	
No	18 (60)
Yes	12 (40)
Admitted to hospital for TB treatment, n = 29	
No	4 (14)
Yes	25 (86)
WHO TB treatment outcome	
Cured	23 (62)
Completed	7 (19)
Failed	1 (3)
Died	4 (11)
Loss to follow-up	2 (5)
Clinical outcome	
Favorable**	30 (81)
Unfavorable††	7 (19)

*EPTB, extrapulmonary tuberculosis; IQR, interquartile range; PTB, pulmonary tuberculosis; TB, tuberculosis; WHO, World Health Organization. †Two cases of EPTB, 1 site specified as urogenital and 1 unspecified. ‡Three cases of PTB and EPTB. Sites of EPTB were peripheral lymph nodes and pleural TB in 1 child and abdominal TB in 2 children. §Severity of TB based on grading of chest radiograph reports available only for 31 children; 2 children had EPTB only and no chest radiograph and 4 children had no chest radiograph despite documented PTB.. ¶Six children were receiving antiretroviral therapy at the start of TB treatment and 1 child started antiretroviral therapy during the course of TB treatment. #Underweight for age (weight-for-age z-score <–2) or as per treating clinician’s report. **Includes all children who were cured or completed treatment. ††Includes all children who failed treatment, died, or were lost to follow-up during treatment.

**Figure 1:**
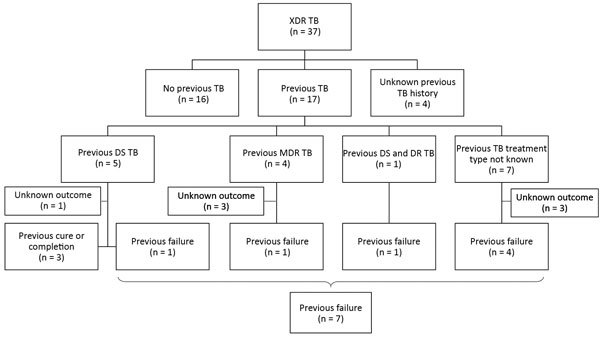
Flow chart for 37 children with confirmed XDR TB and details of TB treatment history, type of TB treatment, and treatment outcome. DR, drug-resistant; DS, drug-susceptible, MDR, multidrug-resistant; TB, tuberculosis; XDR TB, extensively drug-resistant TB.

We also obtained previous TB treatment history for children with XDR TB (Figure 1). Among 33 children who had documented knowledge of previous treatment, 17 (52%) had been previously treated for TB. Of children previously treated, only 10 had known TB treatment outcomes; 7 (70%) had a history of treatment failure.

**Figure 2:**
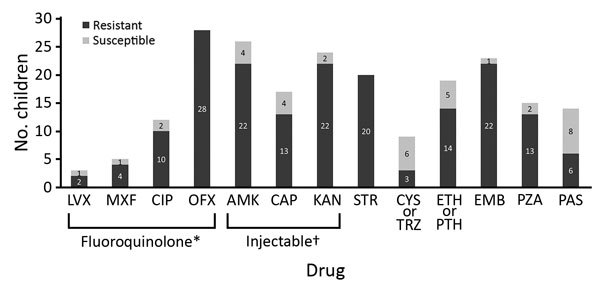
Mycobacterial drug susceptibility test pattern for children treated for extensively drug-resistant tuberculosis. All children had organisms that were resistant to rifampin and isoniazid. *Includes moxifloxacin, levofloxacin, ofloxacin, or ciprofloxacin. †Includes second-line injectable drugs kanamycin, amikacin, or capreomycin. AMK, amikacin, amikacin; CAP, capreomycin; CIP, ciprofloxacin; CYS, cycloserine; EMB, ethambutol; ETH, ethionamide; KAN, kanamycin; LVX, levofloxacin; MXF, moxifloxacin; OFX, ofloxacin; PAS, para-aminosalicylic acid; PTH, prothionamide; PZA, pyrazinamide; STR, streptomycin; TRZ, terizidone.

Data for microbiological investigations were limited. All 37 children had culture-confirmed XDR TB, but only 30 had smear results before treatment, and 10 (33%) were smear positive. Follow-up culture results were infrequently available. The DST pattern for this cohort (Figure 2) showed that all children had isolates with proven resistance to rifampin and isoniazid. Isolates from some children were tested against multiple injectable drugs and fluoroquinolones (some of which were susceptible), but all children in the cohort had isolates that were resistant to >1 second-line injectable drug (kanamycin, amikacin, or capreomycin), and >1 fluoroquinolone (moxifloxacin, levofloxacin, ofloxacin, or ciprofloxacin). DST for additional drugs was performed only for a limited number of children.

**Figure 3:**
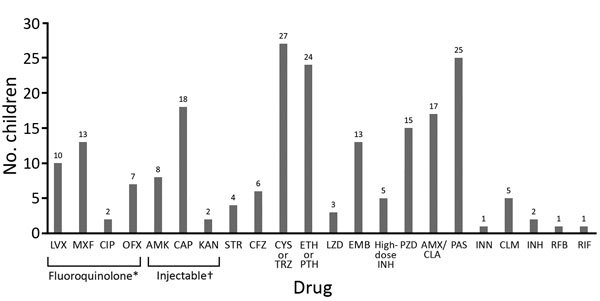
Drugs used for treatment of children with extensively drug resistant tuberculosis. *Includes moxifloxacin, levofloxacin, ofloxacin or ciprofloxacin. †Includes second-line injectable drugs kanamycin, amikacin, or capreomycin. AMK, amikacin, AMX, amoxicillin; CAP, capreomycin; CFZ, clofazimine; CIP, ciprofloxacin; CLA, clavulanic acid; CLM, clarithromycin; CYS, cycloserine; EMB, ethambutol; ETH, ethionamide; INH, isoniazid; INN, thioacetazone; KAN, kanamycin; LVX, levofloxacin; LZD, linezolid; MXF, moxifloxacin; OFX, ofloxacin; PAS, para-aminosalicylic acid; PTH, prothionamide; PZA, pyrazinamide; RFB, fifabutin; RIF, rifampin; STR, streptomycin; TRZ, terizidone.

We also evaluated drugs used to treat these children (Figure 3). No children received bedaquiline or delamanid because both drugs became available only after the study period. The most commonly used drugs were an injectable (n = 27), a fluoroquinolone (n = 26), cycloserine/terizidone (n = 27), ethionamide/prothionamide (n = 24), and para-aminosalicylic acid (n = 25). Capreomycin was the most commonly used injectable drug (Figure 3); for some children, >1 injectable was used sequentially. Of 26 children who received a fluoroquinolone, 13 were given moxifloxacin alone, and 4 were switched between moxifloxacin and other fluoroquinolones. Limited information on timing and reason for changing drugs was available. Duration of use of individual drugs was recorded for a limited number of children, and estimates of individual treatment duration per drug could not be made. Median duration of the intensive phase for 26 children who completed treatment was 7 months (IQR 6.0–8.2 months), and median duration for 23 children who completed the continuation phase was 12.2 months (IQR 10.0–16.2 months).

**Figure 4:**
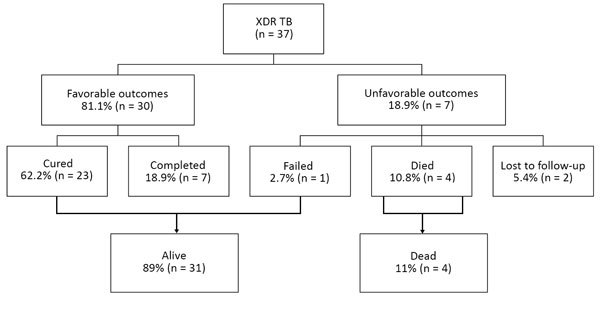
Treatment outcomes for 37 children treated for XDR TB. XDR TB, extensively drug-resistant tuberculosis.

Overall, 30 (81%) children had favorable treatment outcomes (Figure 4). Four (11%) children died during XDR TB treatment; 2 were HIV-infected and receiving antiretroviral therapy. One child (3%) had documentation of treatment failure (HIV infected when receiving antiretroviral therapy) and 2 (5%) were lost to follow-up during treatment (HIV uninfected). We obtained detailed demographic and clinical variables for the 7 children with unfavorable outcomes, including the DST pattern of the isolate and drugs used during treatment (Table 3).

**Table 3:** Demographic and clinical characteristics of 7 children with unfavorable treatment outcomes for XDR TB*

Child	Year of treatment	Age, y/sex†	Weight, kg, at start of XDR TB treatment	HIV status	History of TB	Previous TB outcome	Disease site	Chest radiograph result‡	Drugs to which isolate was resistant	Drugs used at any stage during XDR TB treatment	Outcome
1	2008	9/F	23	–	No	No	PTB	Normal	INH, RIF, OFX, AMK, STR, EMB	INH, CAP, EMB, PZA, ETH, PAS	Lost to follow-up
2	2008	14/F	41	–	U, DST	Failure	PTB	Nonsevere typical TB	INH, RIF, OFX, AMK, STR	Not specified	Lost to follow-up
3	2007	6/M	20	+	No	No	PTB	Nonsevere typical TB	INH, RIF, CIP, AMK, STR, EMB	AMK, EMB, PZA, ETH, CIP	Died
4	2007	14/M	42	+	DS TB	No	PTB, EPTB	Severe not typical TB	INH, RIF, OFX, KAN, STR, EMB, ETH, PAS	CAP, MXF, LVX, ETH, CYS, PAS, AMX, CLA	Died
5	2001	13/F	U	U	DR TB	Failure	PTB	Nonsevere typical TB	INH, RIF, LVX, CIP, KAN, AMK, CAP, STR, EMB, PZA, ETH, PAS	CAP, STR, LVX, CIP, ETH, CYS, PAS, AMX, CLA	Died
6	2009	12/F	19.5	+	Both	Failure	PTB	Nonsevere typical TB	INH, RIF, OFX, KAN	CAP, MXF, EMB, PZA, ETH, CYS, PAS, AMX, CLA, CLM	Failure
7	2013	13/F	34	–	U	No	PTB	U	INH, RIF, MXF, OFX, KAN, AMK, CAP, STR, EMB, PZA, ETH	CAP, LVX, PZA, ETH, CYS, PAS	Died

*Ethionamide or prothionamide was not differentially recorded; where ethionamide is stated, it implies that 1 of the 2 dugs was used. Cycloserine or terizidone was not differentially recorded; where cycloserine is stated, it implies that 1 of the 2 drugs was used. AMK, amikacin; AMX, amoxicillin; CAP, capreomycin; CIP, ciprofloxacin; CLA, clavulanic acid; CLM, clarithromycin; CYS, cycloserine; DS, drug sensitive; DR, drug resistant; DST, drug susceptibility testing; EMB, ethambutol; EPTB, extrapulmonary tuberculosis; ETH, ethionamide; INH, isoniazid; KAN, kanamycin; LVX, levofloxacin; LZD, linezolid; MXF, moxifloxacin; OFX, ofloxacin; PAS, para-aminosalicylic acid; PTB, pulmonary tuberculosis; PZA, pyrazinamide; RIF, rifampin; STR, streptomycin; TB, tuberculosis; U, unknown; XDR TB, extensively drug-resistant tuberculosis; –, negative; +, positive.  †Age at year of diagnosis. ‡By adapted Wiseman classification.

We detected no associations between recorded variables and unfavorable treatment outcome by using univariate analyses. Although HIV-infected children had a 14.3 times (95% CI 1.2–174.8 times) greater odds of an unfavorable outcome than HIV-uninfected children, this logistic regression model used infected, uninfected, and unknown as 3 possible categories of HIV, and differences in the overall model were not significant (p = 0.098). Multivariable analysis was not completed because of lack of significance in all univariate analyses.

## Discussion

Data for treatment of XDR TB in children are limited. Our group of 37 confirmed cases over 15 years represents a small sample yet was larger than those in previous reports, highlighting the serious underdiagnosis and underreporting of XDR TB in children in the published literature. We included data for clinical manifestations, treatment, and outcomes for these children. Overall, we describe encouraging rates of successful treatment outcomes in this small cohort of children despite the limited drug options available to treat children in most instances and the notable lack of access to novel drugs, such as bedaquiline and delamanid, at the time of treatment. All children had confirmed XDR TB; more work is needed to include children given a diagnosis of probable XDR TB in the absence of bacteriological confirmation. Most children were >5 years of age and had pulmonary TB and severe TB. Half of the children had been previously given treatment for TB, and of those children, half had been previously treated with a drug-susceptible TB treatment regimen.

Of 975 children described in the MDR TB IPD, only 37 had confirmed XDR TB, which might reflect the limited access to second-line DST. The older median age (11 years) observed in this group than that for a pediatric MDR TB IPD review (median age 7.1 years) (*6*) might indicate that younger children might not have been as likely to have been investigated and given a diagnosis of XDR TB. Because it has been estimated that 80% of childhood TB deaths occur in children <5 years of age (*21*), death before diagnosis and treatment initiation in the youngest children might also contribute to the older age of this cohort. Another potential explanation is that younger children might have been more likely to have been given treatment without microbiological confirmation (given a diagnosis based only on clinical findings) because of their typical paucibacillary disease and challenges in obtaining respiratory specimens. Finally, because older children are more likely to have adult-type TB with higher bacillary burden, bacteriological confirmation is more likely (*22*).

It is essential that empiric treatment for XDR TB in children be based on DST of the organism of the putative TB source case-patient, but the need for adequate sampling for TB bacteriology including gastric aspirates, or other samples, cannot be overstated (*23*–*25*). If one considers the risk for rapid disease progression (*26*), treatment initiation in children should not be delayed. Although DST for the XDR TB isolate might be used for development of a targeted treatment regimen, a good contact history with DST of the organism of the source case-patient might also be used where confirmation is not possible. In our study, 28 (76%) children had recorded information on source cases. Of these children, 20 (70%) had an identified source case during their episode of TB, but we did not have sufficient information to define the DST pattern of the source case. This finding highlights the need for contact investigation of source cases. Early screenings of households after diagnosis of TB is an opportunity to identify at-risk contacts. Symptom screening and sampling of child contacts could facilitate earlier management.

*M. tuberculosis* DST patterns in this cohort were limited because the review spanned 15 years starting in 1999. Determination of fluoroquinolone resistance varied and, for the definition we applied in this study, ciprofloxacin resistance was included. Data for additional testing of drugs was limited and did not enable us to make inferences about the DST pattern and the drug or regimen chosen or any changes in DST pattern during the course of treatment.

Although we were not able to evaluate the role of specific drugs because of the small number of children and the variability in treatment regimens, we found patterns of commonly used drugs. Most (n = 27) children received an injectable drug, and although more exact data were limited, the median duration used was 7 months. Isolates from all children had confirmed resistance to an injectable drug; thus, it is a concern that an injectable drug was used for so many children, particularly given the risk for adverse events, including permanent hearing loss (*27**,**28*). It is possible that clinicians expected incomplete cross-resistance within the injectable drug group, a rationale supported by the predominance of capreomycin use (*29*). However, cross-resistance within this group is relatively common (*30*). It is also possible that children might have been given these agents before second-line DST results became available. *M. tuberculosis* strains resistant to earlier-generation fluoroquinolones might retain susceptibility to a later-generation fluoroquinolone (*31*), which might have supported fluoroquinolone use despite documented resistance. Most (n = 26) children received a fluoroquinolone, and this drug was a later-generation treatment for >50% of case-patients. This finding might reflect the anticipation of clinicians for some activity of the drug, despite DST results and limited other treatment options.

Most children in our cohort were given treatment before availability of new drugs. Linezolid was only used for 3 children and clofazimine for 6 children, whereas bedaquiline and delamanid were not used. Para-aminosalicylic acid, ethionamide or prothionamide, and cycloserine or terizidone were used frequently, highlighting the limited drug options available and the necessity to use drugs with major toxicity and relatively poor efficacy. With new drugs, alternatives for children with drug-resistant TB are being explored. The WHO endorsement of delamanid for older children (>6 years of age) with MDR TB (*32*) and early reports on the use of bedaquiline in older children (>10 years of age) (*33*) might be initial steps in finding the optimum treatment regimen for XDR TB in children.

Children with confirmed XDR TB had similar successful outcomes (81%) as children given treatment for clinically diagnosed and bacteriologically confirmed MDR TB (78%) over the same study period (*6*). Although loss to follow-up was lower (5.4% for XDR TB and 11.2% for MDR TB) (*6*), our cohort of children with XDR TB was smaller, and children with XDR TB might have had more intensive follow-up than children with MDR TB. WHO combined program data for 6,777 XDR TB case-patients (adults and children) reported from 52 countries during 2014 found treatment success in only 30%, with 28% deaths, 21% treatment failures, and 20% lost to follow-up (*2*). Overall mortality rates exceeded 40% in India and South Africa (*2*). High mortality rates for XDR TB in adults has been well documented (*34*–*37*). In our cohort of children with XDR TB, 4 (10.8%) died during treatment.

Considering the natural history of TB, we acknowledge that spontaneous cure in children occurs and might also contribute to the good outcomes seen. We note that none of the children included in our cohort had TB meningitis, which is well known to have poor outcomes (*38*), and this finding might contribute to the good outcomes we report. This lower mortality rate might be caused by this group representing a survival bias, only including children who were alive to make the diagnosis and initiate treatment. In addition, children tend to have lower organism load (paucibacillary), have less concomitant pathology, and be treated with more tailored individualized regimens compared with adults, which might improve survival. Diagnosis and treatment of XDR TB was historically limited to tertiary centers or specialized centers of excellence for TB care, providing more resources for care and improving diagnosis and treatment outcomes. Most (86%) children were admitted to a hospital and would have likely had good adherence support. We acknowledge that this cohort consisted of a small number of selected children and are therefore cautious about generalizability.

A further limitation of our study is poor reporting of adverse events, which could not be analyzed. Poor adverse event reporting has been identified by WHO as a problem (*2*). Systematic monitoring of TB drug safety for children is crucial, especially if one considers introduction of new drugs. Given the modest sample size and the limitations regarding the dose and exact duration of drugs, we could not analyze the effect of individual drugs, regimen combinations, or duration of treatment on final treatment outcomes. Newer drugs require evaluation because WHO has recommended use of delamanid for children and adolescents (*32*).

Access to newer TB drugs and effective shorter MDR TB regimens is improving. Systematic reviews of MDR TB or XDR TB have identified the benefits of linezolid, a larger number of effective drugs, the number of drugs used in each phase of treatment, and the duration of treatment (*39*–*42*). Although there might be some indication for use of more drugs during the intensive phase and continuation phase, with longer duration of treatment for XDR TB, this evidence is still limited (*41*). Studies to date have mainly included adults (*39*–*42*). Our findings highlight the need for more studies evaluating new drugs and treatment regimens in children with XDR TB.

In conclusion, we report treatment of XDR TB for children spanning 15 years. The limited number of children identified highlights a major gap in diagnosing and reporting XDR TB in children. The high proportion of favorable treatment outcomes and considerably lower mortality rates compared with those for adults is encouraging. We found considerable variability of regimens used and duration of treatment in children, but this review preceded availability and use of linezolid, clofazimine, bedaquiline, and delamanid. More collaborative, multicenter prospective cohorts are needed to collect better and more extensive data for children with drug-resistant TB. Evaluation of shorter effective and safe regimens for children with XDR TB is urgently needed.

## References

[1] World Health Organization. Extensively drug-resistant tuberculosis (XDR-TB): recommendations for prevention and control. Wkly Epidemiol Rec. 2006;81:430–2. 17096498

[2] World Health Organization. Global Tuberculosis Report 2017. Geneva: The Organization; 2017.

[3] Jenkins HE, Tolman AW, Yuen CM, Parr JB, Keshavjee S, Pérez-Vélez CM, et al. Incidence of multidrug-resistant tuberculosis disease in children: systematic review and global estimates. Lancet. 2014;383:1572–9. 10.1016/S0140-6736(14)60195-1 24671080 PMC4094366

[4] Dodd PJ, Sismanidis C, Seddon JA. Global burden of drug-resistant tuberculosis in children: a mathematical modelling study. Lancet Infect Dis. 2016;16:1193–201. 10.1016/S1473-3099(16)30132-3 27342768

[5] World Health Organization. WHO treatment guidelines for drug-resistant tuberculosis. Geneva: The Organization; 2016.

[6] Harausz EP, Garcia-Prats AJ, Law S, Schaaf HS, Kredo T, Seddon JA, et al.; Collaborative Group for Meta-Analysis of Paediatric Individual Patient Data in MDR-TB. Treatment and outcomes in children with multidrug-resistant tuberculosis: A systematic review and individual patient data meta-analysis. PLoS Med. 2018;15:e1002591. 10.1371/journal.pmed.1002591 29995958 PMC6040687

[7] World Health Organization. Guidance for national tuberculosis programmes on the management of tuberculosis in children. 2nd ed. Geneva: The Organization; 2014. 24999516

[8] Wiseman CA, Gie RP, Starke JR, Schaaf HS, Donald PR, Cotton MF, et al. A proposed comprehensive classification of tuberculosis disease severity in children. Pediatr Infect Dis J. 2012;31:347–52. 10.1097/INF.0b013e318243e27b 22315002

[9] World Health Organization. Definitions and reporting framework for tuberculosis. 2013 revision. Geneva: The Organization; 2014.

[10] Banerjee R, Allen J, Westenhouse J, Oh P, Elms W, Desmond E, et al. Extensively drug-resistant tuberculosis in california, 1993-2006. Clin Infect Dis. 2008;47:450–7. 10.1086/590009 18616396

[11] Drobac PC, Mukherjee JS, Joseph JK, Mitnick C, Furin JJ, del Castillo H, et al. Community-based therapy for children with multidrug-resistant tuberculosis. Pediatrics. 2006;117:2022–9. 10.1542/peds.2005-2235 16740844

[12] Gegia M, Jenkins HE, Kalandadze I, Furin J. Outcomes of children treated for tuberculosis with second-line medications in Georgia, 2009-2011. Int J Tuberc Lung Dis. 2013;17:624–9. 10.5588/ijtld.12.0792 23575328 PMC3734931

[13] Hicks RM, Padayatchi N, Shah NS, Wolf A, Werner L, Sunkari VB, et al. Malnutrition associated with unfavorable outcome and death among South African MDR-TB and HIV co-infected children. Int J Tuberc Lung Dis. 2014;18:1074–83. 10.5588/ijtld.14.0231 25189555

[14] Isaakidis P, Paryani R, Khan S, Mansoor H, Manglani M, Valiyakath A, et al. Poor outcomes in a cohort of HIV-infected adolescents undergoing treatment for multidrug-resistant tuberculosis in Mumbai, India. PLoS One. 2013;8:e68869. 10.1371/journal.pone.0068869 23894358 PMC3716893

[15] Kuksa L, Riekstina V, Leimane V, Ozere I, Skenders G, Van den Bergh R, et al. Multi- and extensively drug-resistant tuberculosis in Latvia: trends, characteristics and treatment outcomes. Public Health Action. 2014;4(Suppl 2):S47–53. 10.5588/pha.14.0041 26393098 PMC4547513

[16] Smirnova PA, Turkova A, Nikishova EI, Seddon JA, Chappell E, Zolotaya OA, et al. Multidrug-resistant tuberculosis in children in northwest Russia: an observational cohort study. Eur Respir J. 2016;48:1496–9. 10.1183/13993003.00354-2016 27587542

[17] Moore BK, Anyalechi E, van der Walt M, Smith S, Erasmus L, Lancaster J, et al. Epidemiology of drug-resistant tuberculosis among children and adolescents in South Africa, 2005-2010. Int J Tuberc Lung Dis. 2015;19:663–9. 10.5588/ijtld.14.0879 25946356 PMC4886335

[18] Seddon JA, Hesseling AC, Willemse M, Donald PR, Schaaf HS. Culture-confirmed multidrug-resistant tuberculosis in children: clinical features, treatment, and outcome. Clin Infect Dis. 2012;54:157–66. 10.1093/cid/cir772 22052896

[19] Seddon JA, Hesseling AC, Godfrey-Faussett P, Schaaf HS. High treatment success in children treated for multidrug-resistant tuberculosis: an observational cohort study. Thorax. 2014;69:458–64. 10.1136/thoraxjnl-2013-203900 24064441

[20] Swaminathan A, du Cros P, Seddon JA, Quinnell S, Bobokhojaev OI, Dusmatova Z, et al. Treating children for drug-resistant tuberculosis in Tajikistan with Group 5 medications. Int J Tuberc Lung Dis. 2016;20:474–8. 10.5588/ijtld.15.0666 26970156

[21] Dodd PJ, Yuen CM, Sismanidis C, Seddon JA, Jenkins HE. The global burden of tuberculosis mortality in children: a mathematical modelling study. Lancet Glob Health. 2017;5:e898–906. 10.1016/S2214-109X(17)30289-9 28807188 PMC5556253

[22] Marais BJ, Gie RP, Schaaf HS, Hesseling AC, Enarson DA, Beyers N. The spectrum of disease in children treated for tuberculosis in a highly endemic area. Int J Tuberc Lung Dis. 2006;10:732–8. 16848333

[23] Marcy O, Ung V, Goyet S, Borand L, Msellati P, Tejiokem M, et al. Performance of Xpert MTB/RIF and alternative specimen collection methods for the diagnosis of tuberculosis in HIV-infected children. Clin Infect Dis. 2016;62:1161–8. 10.1093/cid/ciw036 26908804

[24] Zar HJ, Workman L, Isaacs W, Munro J, Black F, Eley B, et al. Rapid molecular diagnosis of pulmonary tuberculosis in children using nasopharyngeal specimens. Clin Infect Dis. 2012;55:1088–95. 10.1093/cid/cis598 22752518 PMC3529610

[25] Detjen AK, Walters E. Editorial commentary: improving children’s access to new tuberculosis d iagnostic tools starts with the collection of appropriate specimens. Clin Infect Dis. 2016;62:1169–71. 10.1093/cid/ciw042 26908805

[26] Marais BJ, Gie RP, Schaaf HS, Hesseling AC, Obihara CC, Starke JJ, et al. The natural history of childhood intra-thoracic tuberculosis: a critical review of literature from the pre-chemotherapy era. Int J Tuberc Lung Dis. 2004;8:392–402. 15141729

[27] Seddon JA, Thee S, Jacobs K, Ebrahim A, Hesseling AC, Schaaf HS. Hearing loss in children treated for multidrug-resistant tuberculosis. J Infect. 2013;66:320–9. 10.1016/j.jinf.2012.09.002 22960077

[28] Seddon JA, Godfrey-Faussett P, Jacobs K, Ebrahim A, Hesseling AC, Schaaf HS. Hearing loss in patients on treatment for drug-resistant tuberculosis. Eur Respir J. 2012;40:1277–86. 10.1183/09031936.00044812 22700838

[29] Zhang Z, Liu M, Wang Y, Pang Y, Kam KM, Zhao Y. Molecular and phenotypic characterization of multidrug-resistant *Mycobacterium tuberculosis* isolates resistant to kanamycin, amikacin, and capreomycin in China. Eur J Clin Microbiol Infect Dis. 2014;33:1959–66. 10.1007/s10096-014-2144-5 24906439

[30] Schaaf HS, Seddon JA, Caminero JA. Second-line antituberculosis drugs: current knowledge, recent research findings and controversies. Progr Respir Res. 2011;40:81–95. 10.1159/000324382

[31] Kam KM, Yip CW, Cheung TL, Tang HS, Leung OC, Chan MY. Stepwise decrease in moxifloxacin susceptibility amongst clinical isolates of multidrug-resistant *Mycobacterium tuberculosis*: correlation with ofloxacin susceptibility. Microb Drug Resist. 2006;12:7–11. 10.1089/mdr.2006.12.7 16584301

[32] World Health Organization. The use of delamanid in the treatment of multidrug-resistant tuberculosis in children and adolescents: interim policy guidance. Geneva: The Organization; 2016. 27854402

[33] Achar J, Hewison C, Cavalheiro AP, Skrahina A, Cajazeiro J, Nargiza P, et al. Off-label use of bedaquiline in children and adolescents with multidrug-resistant tuberculosis. Emerg Infect Dis. 2017;23:1711–3. 10.3201/eid2310.170303 28758889 PMC5621552

[34] Gandhi NR, Moll A, Sturm AW, Pawinski R, Govender T, Lalloo U, et al. Extensively drug-resistant tuberculosis as a cause of death in patients co-infected with tuberculosis and HIV in a rural area of South Africa. Lancet. 2006;368:1575–80. 10.1016/S0140-6736(06)69573-1 17084757

[35] Gandhi NR, Andrews JR, Brust JC, Montreuil R, Weissman D, Heo M, et al. Risk factors for mortality among MDR- and XDR-TB patients in a high HIV prevalence setting. Int J Tuberc Lung Dis. 2012;16:90–7. 10.5588/ijtld.11.0153 22236852 PMC3302205

[36] Dheda K, Shean K, Zumla A, Badri M, Streicher EM, Page-Shipp L, et al. Early treatment outcomes and HIV status of patients with extensively drug-resistant tuberculosis in South Africa: a retrospective cohort study. Lancet. 2010;375:1798–807. 10.1016/S0140-6736(10)60492-8 20488525

[37] Pietersen E, Ignatius E, Streicher EM, Mastrapa B, Padanilam X, Pooran A, et al. Long-term outcomes of patients with extensively drug-resistant tuberculosis in South Africa: a cohort study. Lancet. 2014;383:1230–9. 10.1016/S0140-6736(13)62675-6 24439237

[38] Chiang SS, Khan FA, Milstein MB, Tolman AW, Benedetti A, Starke JR, et al. Treatment outcomes of childhood tuberculous meningitis: a systematic review and meta-analysis. Lancet Infect Dis. 2014;14:947–57. 10.1016/S1473-3099(14)70852-7 25108337

[39] Zhang X, Falagas ME, Vardakas KZ, Wang R, Qin R, Wang J, et al. Systematic review and meta-analysis of the efficacy and safety of therapy with linezolid containing regimens in the treatment of multidrug-resistant and extensively drug-resistant tuberculosis. J Thorac Dis. 2015;7:603–15. 25973226 10.3978/j.issn.2072-1439.2015.03.10 PMC4419320

[40] Ahuja SD, Ashkin D, Avendano M, Banerjee R, Bauer M, Bayona JN, et al.; Collaborative Group for Meta-Analysis of Individual Patient Data in MDR-TB. Multidrug resistant pulmonary tuberculosis treatment regimens and patient outcomes: an individual patient data meta-analysis of 9,153 patients. PLoS Med. 2012;9:e1001300. 10.1371/journal.pmed.1001300 22952439 PMC3429397

[41] Falzon D, Gandhi N, Migliori GB, Sotgiu G, Cox HS, Holtz TH, et al.; Collaborative Group for Meta-Analysis of Individual Patient Data in MDR-TB. Resistance to fluoroquinolones and second-line injectable drugs: impact on multidrug-resistant TB outcomes. Eur Respir J. 2013;42:156–68. 10.1183/09031936.00134712 23100499 PMC4487776

[42] Migliori GB, Sotgiu G, Gandhi NR, Falzon D, DeRiemer K, Centis R, et al.; “The Collaborative Group for Meta-Analysis of Individual Patient Data in MDR-TB”. Drug resistance beyond extensively drug-resistant tuberculosis: individual patient data meta-analysis. Eur Respir J. 2013;42:169–79. 10.1183/09031936.00136312 23060633 PMC4498806

